# Molecular Basis of Plant Stress Tolerance: Current Status and Future Perspectives

**DOI:** 10.3390/cimb47110918

**Published:** 2025-11-05

**Authors:** Sajid Ali

**Affiliations:** Department of Horticulture and Life Science, Yeungnam University, Gyeongsan 38541, Republic of Korea; drsajid@yu.ac.kr

## 1. Introduction

I write this interim editorial to record my heartfelt appreciation to all participants and to express sincere gratitude to the authors, reviewers, and editorial staff of *Current Issues in Molecular Biology*, whose intellectual engagement and professional dedication have been pivotal to the success of my Special Issue “*Molecular Mechanisms in Plant Stress Tolerance*”.

Plants are continuously exposed to a spectrum of biotic and abiotic stressors, including drought, salinity, extreme temperatures, heavy metals, and pathogen invasion [1,2]. As sessile organisms, they have evolved highly coordinated molecular systems to perceive environmental cues and orchestrate adaptive responses [3,4]. Plant stress biology is transitioning from lists of parts to a clear picture of how signals work together. When plants face drought, heat, or pathogens, they integrate redox cues from reactive oxygen species (ROS) and reactive nitrogen species (RNS), carefully timed calcium ion (Ca^2+^) pulses, and phosphorylation cascades driven by mitogen-activated protein kinases (MAPKs), calcium-dependent protein kinases (CDPKs), and the calcineurin B-like-CIPK system (CBL-CIPK). These layers interact with hormone pathways, abscisic acid (ABA), salicylic acid (SA), jasmonic acid (JA), and ethylene (ET), while transcription factors translate the combined signals into gene expression programs that enhance antioxidant defenses, maintain protein quality, produce osmolytes, and regulate ion transport. The outcome is a finely calibrated program that strengthens cellular defenses and restores homeostasis while sustaining growth and development [5,6,7].

Despite significant progress, key questions remain—particularly regarding how concurrent pathways are coordinated and remembered. Many pathways are activated simultaneously, share key components, and can “remember” prior stress, which challenges simple, linear explanations. To close these gaps, we need single-cell and subcellular measurements of Ca^2+^ and ROS/RNS dynamics, quantitative phosphoproteomics linked to causal perturbations, and models that generate testable predictions—all reported with findable, accessible, interoperable, and reusable (FAIR) metadata [8]. With this network-aware, quantitative approach, breeders and biotechnologists can more rationally combine traits—modulating hormone sensitivity, antioxidant capacity, and transport processes—to produce climate-resilient crops without compromising yield or quality [9,10,11]. Additionally, epigenetic modifications—including DNA methylation, histone acetylation, and non-coding RNAs—contribute to the establishment of “stress memory,” enabling plants to mount faster and more effective responses to recurrent stresses. Mechanisms that sustain protein homeostasis, such as chaperone-assisted folding, ubiquitin–proteasome degradation, and autophagy, further reinforce cellular resilience needs to be considered [12,13].

Advancements in omics technologies have revolutionized plant stress biology. Transcriptomic and proteomic analyses now enable high-resolution mapping of stress-induced gene networks, while metabolomics provides functional evidence of altered biochemical fluxes [14]. Integrating transcriptomic, proteomic, and metabolomic datasets through systems biology and computational modeling offers a holistic view of the molecular networks governing stress tolerance [15,16]. Multi-omics analyses reveal how genes, metabolites, and proteins interact under combined stresses. Metabolomic and proteomic studies in crops identify flavonoids, and antioxidative enzymes that are essential for stabilization and detoxification. Gene-editing tools, including CRISPR/Cas, have confirmed key regulators that enhance water-use efficiency, photosynthetic stability, and yield. Functional genomics and genome editing approaches have validated several regulators critical under stress conditions [17,18].

Despite these advances, several challenges persist. Translating molecular insights from model systems to crop improvement requires bridging genotype–environment interactions under field conditions. Many molecular discoveries arise from single-stress experiments, whereas real-world environments impose multifactorial stresses that act simultaneously. Priorities include rigorously designed combined- and sequential-stress studies with defined dose, rate, and order, coupled with continuous in situ monitoring via high-throughput phenotyping and remote sensing to connect cellular events (ROS/RNS, Ca^2+^, MAPKs, CDPKs, CBL–CIPK) to organ- and canopy-level performance. Another frontier lies in systems-level integration—linking gene expression, metabolic shifts, and physiological performance through machine learning and network modeling. Such integrative frameworks can identify key regulatory nodes and predict adaptive outcomes. In addition, epigenome engineering and synthetic biology offer new tools to design stress-responsive circuits that dynamically fine-tune gene expression. Finally, sustainable crop improvement demands a translational pipeline that couples molecular breeding with agronomic validation. The integration of omics-based biomarkers, precision editing, and microbiome-based strategies holds promise for achieving resilience in the face of global climate change.
